# Alveolar distraction osteogenesis for dental implant treatments of the vertical bone atrophy: A systematic review

**DOI:** 10.4317/medoral.22750

**Published:** 2018-12-24

**Authors:** Jorge Toledano-Serrabona, Mª Ángeles Sánchez-Garcés, Alba Sánchez-Torres, Cosme Gay-Escoda

**Affiliations:** 1 DDS. Dental Degree. School of Medicine and Health Sciences, University of Barcelona, Barcelona (Spain); 2 MD, DDS, MS, PhD, EBOS. Lecturer in Oral Surgery. Master’s Degree Program in Oral Surgery and Implantology, School of Medicine and Health Sciences, University of Barcelona. Researcher of the IDIBELL institute, Barcelona (Spain); 3 DDS, MS. Associate Professor of Oral Surgery. Master’s Degree Program in Oral Surgery and Implantology, School of Medicine and Health Sciences, University of Barcelona. Researcher of the IDIBELL Institute, Barcelona (Spain); 4 MD, DDS, MS, PhD, EBOS, OMFS. Chairman and Professor of Oral and Maxillofacial Surgery, School of Medicine and Health Sciences, Barcelona. Director of the Master’s Degree Program in Oral Surgery and Implantology (EFHRE International University/FUCSO). Coordinator/Researcher of the IDIBELL Institute. Head of the Oral Surgery, Implantology and Maxillofacial Surgery, Department of the Teknon Medical Center, Barcelona (Spain)

## Abstract

**Background:**

To determine if alveolar vertical distraction osteogenesis obtains better results compared to other bone regeneration treatments (guided bone regeneration with membranes and / or filling material, or autogenous bone graft) in terms of bone gain, complications, and implant survival and success rates.

**Material and Methods:**

An electronic search was performed in Pubmed (MEDLINE), Cochrane Library and Scopus databases in March 2017. Besides, a manual search was carried out. Inclusion criteria were randomized controlled trials published within the last 10 years with at least 1 year of follow-up after implant placement. No language restriction was applied. Exclusion criteria were studies in patients with bone defects produced by trauma, congenital malformation or oncologic surgical treatment. The methodological quality of the selected studies was evaluated by means of the Cochrane Collaboration’s Tool for assessing risk of bias. The reports were classified into different levels of recommendation according to the “Strength of Recommendation Taxonomy “.

**Results:**

Out of 221 articles, two randomized controlled trials were finally selected for the inclusion in the systematic review. Bone gain and complications were higher with the alveolar vertical distraction osteogenesis compared to the autologous bone graft. There was higher bone resorption with the autologous bone graft. Implant survival and success rates were similar between studies, despite of the used technique.

**Conclusions:**

Both alveolar distraction osteogenesis and autogenous bone graft are effective bone regeneration techniques for the treatment of mandibular vertical bone atrophy. A level B recommendation can be established for the use of alveolar vertical distraction osteogenesis for the treatment of the mandibular vertical bone atrophy.

** Key words:**Distraction osteogenesis, alveolar ridge augmentation, alveolar bone loss.

## Introduction

Alveolar bone atrophy is one of the most common issues of oral rehabilitations with dental implants (1). Alveolar bone can be regenerated horizontal and vertically, being the vertical bone atrophy the most challenging to regenerate because of the surgical difficulty, the anatomic limits that may produce a minor vascularization and the need of a hermetic primary closure of the wound (2,3). There are different techniques used for vertical bone augmentation such as guided bone regeneration (GBR), alveolar distraction osteogenesis (ADO) and autogenous bone graft (ABG), among others. All these have shown favorable clinical and histological results (2).

ADO is a bone regeneration technique, introduced by Chin and Toth in 1996 (4) based in a biological process used for regenerate and consolidate bone between two bone segments obtained after osteotomy. These segments have been gradually separated by the process of distraction (5). ADO can be performed both horizontally (AHDO) and vertically (AVDO) (6,7).

Even though the AVDO has shown good results in clinical studies (8-11), there are few articles comparing other bone regeneration techniques and evaluating bone gain and outcomes of dental implants at the long-term.

The aim of this systematic review was to gather the available scientific evidence to answer the PICO question (12): “¿In healthy patients with mandibular vertical bone atrophy who need bone regeneration prior to placing dental implants, does alveolar vertical distraction osteogenesis obtain better results compared to other bone regeneration treatments (guided bone regeneration with membranes and / or filling material, or autogenous bone graft) in terms of bone gain, complications, and implant survival and success rates?”.

## Material and Methods

This systematic review has been performed according to “Preferred Reporting Items for Systematic Reviews and Meta-Analyses” (PRISMA) guidelines (12).  Table 1 shows the individual parts of the PICO question.

**Table 1:**
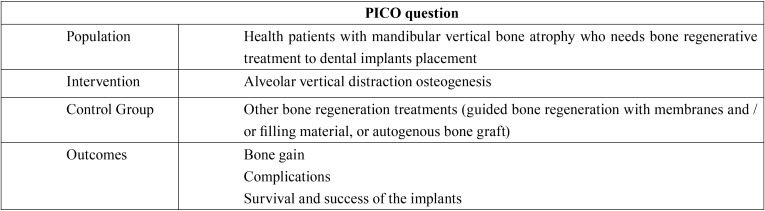
PICO question: P= population; I= intervention; C= control group; O= outcomes.

Inclusion criteria were randomized controlled trials comparing AVDO prior to implant placement in patients with mandibular vertical bone atrophy to other regenerative techniques (GBR with membranes and/or filling material, or ABG) in terms of bone gain (mm), complications, and implant survival and success rates, with at least 1 year of follow-up. The included studies have to be published during the last 10 years; no language restriction was applied. Exclusion criteria were studies about bone regeneration in patients with bone defects produced by traumatism, congenital malformations or oncologic surgical treatment.

Two independent reviewers (JTS and AST) conducted an electronic search in Pubmed (MEDLINE), Cochrane Library and Scopus databases in March 2017. The search strategy was (“Osteogenesis, Distraction”[Mesh] OR “distraction osteogenesis” OR “alveolar distraction” OR “alveolar vertical distraction” OR “vertical alveolar ridge distraction” OR “alveolar vertical distraction osteogenesis” AND “Alveolar Ridge Augmentation”[Mesh] OR “vertical ridge augmentation” OR “vertical ridge regeneration” OR “vertical bone augmentation” AND “Alveolar Bone Loss”[Mesh] OR “atrophic jaws”). First, they selected articles by title and abstract and finally, by reading the full-text of relevant articles to include them in the systematic review. Any disagreement regarding inclusion was resolved by discussion between the two investigators. Besides, a manual search of articles published during the last 10 years was performed in the following journals: The International Journal of Oral and Maxillofacial Implants, Clinical Oral Implants Research, Periodontology 2000, Journal of Clinical Periodontology, Journal of Periodontology, Journal of Periodontal Research and Clinical Oral Investigations to identify the articles not included in the results of the electronic search.

A flow chart summarizing the search process was made according to PRISMA guidelines. The selected articles were classified into different levels of evidence following SORT criteria (13). Furthermore, the risk of bias of each article was determined with the “Cochrane Handbook for Systematic Reviews of Interventions, version 5.1.0” (14).

Finally, a qualitative synthesis of the results of the included studies was performed and displayed in Tables. The registered variables were the total number of implants and patients, distractor type, donor site, the vertical bone gain (mm), bone remodeling (mm), implant survival and success rate, postoperative complications and follow-up time (months).

Results

**Figure 1:**
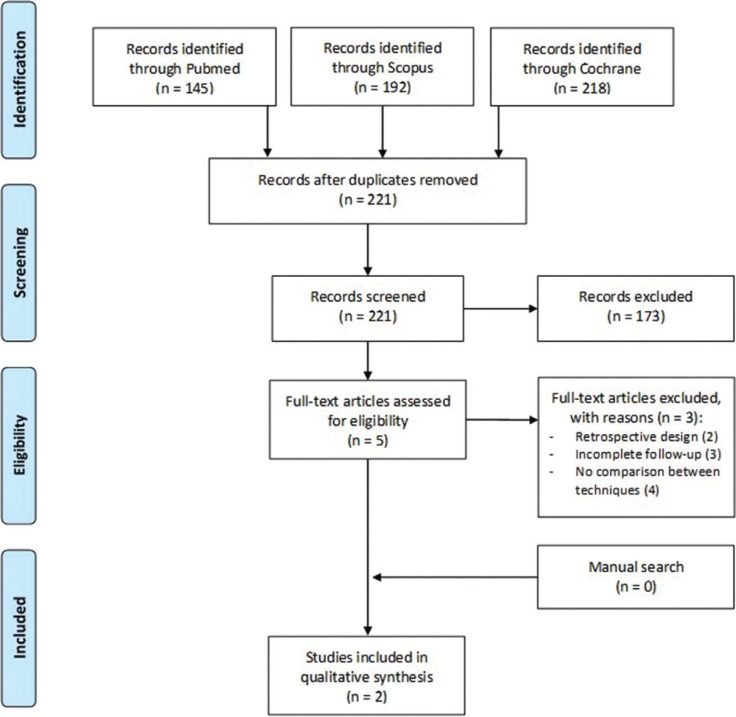
PRISMA flow chart of the study selection process.

Figure 1 shows the flow chart of the selected articles through the systematic review process according to PRISMA guidelines. The initial electronic search yielded 555 articles and, after the exclusion of duplicates, a total of 221 citations reminded. After reading titles and abstracts, 5 articles were selected for the full-text evaluation. Three publications were excluded after applying study criteria: one was removed because of a retrospective design (15), one was excluded as the insufficient follow-up (16) and no comparison between AVDO and different bone regeneration techniques (17). Finally, 2 studies were included in the systematic review (18,19). A meta-analysis was not performed due to the heterogeneity of the selected studies. The level of agreement between reviewers was excellent, with a kappa index of 1.

The 2 articles selected involved 28 patients with 77 implants. 27 patients had unilateral and only 1 had bilateral treated mandibular vertical bone atrophy. Specifically, 14 patients were treated by and AVDO and 14 by ABG.

**Figure 2:**
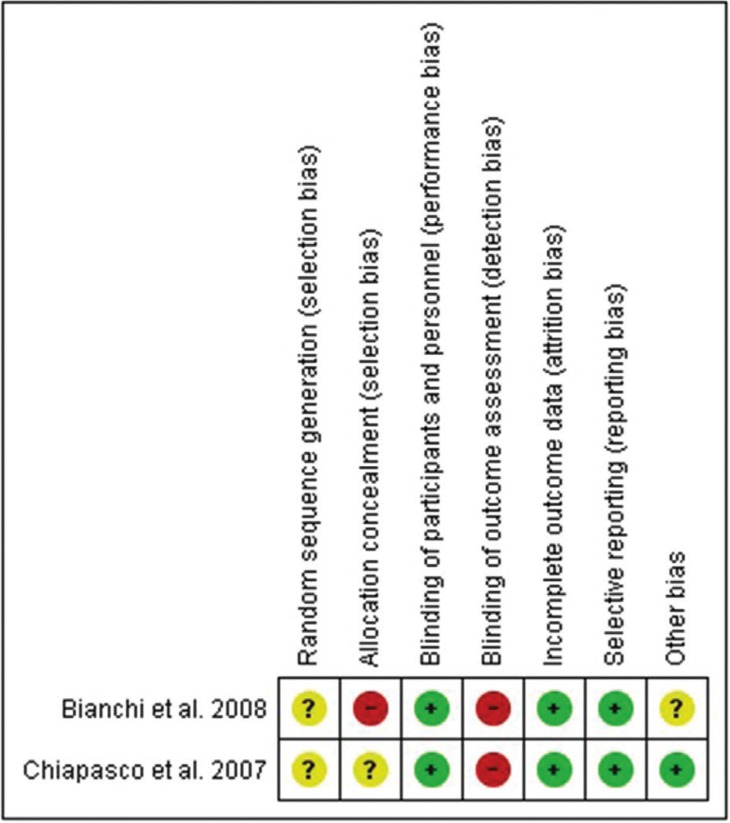
The Cochrane Collaboration’s tool for assessing risk of bias for randomized controlled trials.

As shown in Figure 2, both articles were classified as having high risk of bias due to the lack of allocation concealment (18) and blinding of outcome assessment (18,19). No details regarding to the method used for generating the random sequence appeared in the selected articles. Moreover, an unclear risk of bias was considered by the authors in “other bias” for Bianchi *et al.* (18) as distinct types of distractors and implants were used and this may influence the obtained results. Included articles have a level 2 quality according to SORT criteria, as they are RCTs with limited quality.

 Table 2 shows the characteristics and main results of the studies included in this systematic review. Bianchi *et al.* (18) found statistically significant differences for bone gain in AVDO group compared to ABG from iliac crest, although with significantly more postoperative complications in AVDO group. However, Chiapasco *et al.* (19) found similar bone gain for both groups and significantly more bone resorption prior to implant placement in ABG group, from mandibular ramus origin.

**Table 2:**
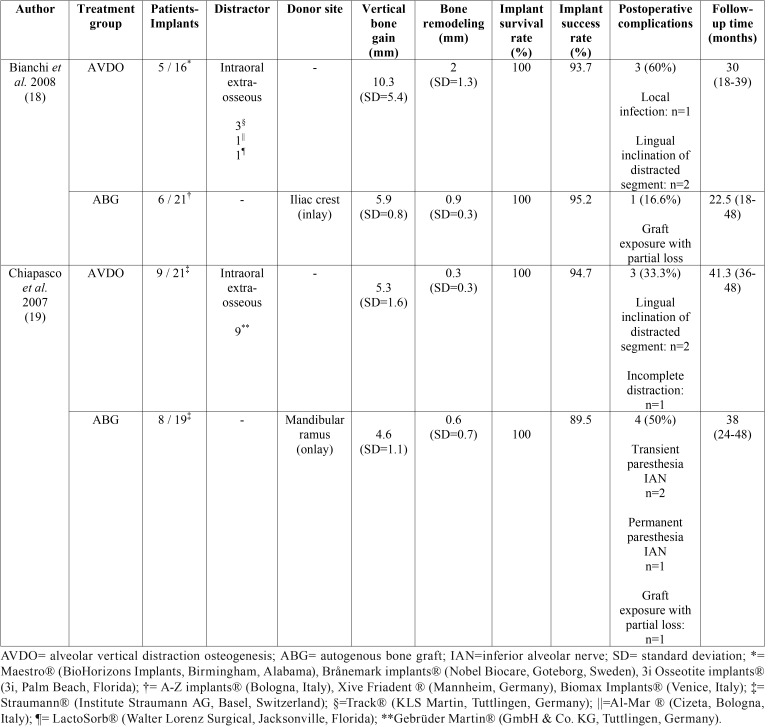
Characteristics of the studies included in the systematic review.

Implant survival rate was 100% for both groups from each study. The success rate for implants placed in ABG group ranged from 89.5 to 95.2% after 18 to 48 months of follow-up. Similarly, the success rate for implants belonging to AVDO group varied from 93.7 to 94.7% within the same follow-up time. Both studies used criteria proposed by Albrektsson *et al.* (20), categorizing the failure of a dental implant as a peri-implant bone loss greater than 1.5 mm during the first year after loading. Thus, no relevant differences between studies were found for implant survival and success rates.

## Discussion

This systematic review has compared the results obtained with AVDO and ABG. No other comparisons could be performed due to the lack of studies comparing AVDO with other bone regeneration techniques for the treatment of mandibular vertical bone atrophy. Bone gain after the surgical augmentation and bone remodeling prior to implant placement seems to have controversial values, as Bianchi *et al.*(18) found more bone gain in the AVDO group although Chiapasco *et al.* (19) did not find relevant differences.

From the need to repair the dentoalveolar bone defects, different materials and bone regeneration techniques have been developed. The types of bone grafts are classified in ABG, allografts, xenografts and synthetic grafts (21). ABG remains the gold standard due its osteogenic and non-immunogenic potential (22). However, the inconvenient of this technique is the high morbidity due to the need of a second surgical field (10,23,24).

Grafts can be harvested from intraoral and extraoral sites, having different types of ossification. The mandibular ramus has an intramembranous origin which means less bone resorption after healing when compared to endochondral bones. In addition, the graft harvesting can be performed in the same surgery and under local anesthesia (25). On the contrary, iliac crest is an endochondral bone and it displays a more complete graft resorption during healing although this area can provide larger blocks than intraoral autogenous bone grafts (26). Despite of that, selected studies (18,19) showed similar results for bone resorption of ABGs. Greater differences were found for bone remodeling in AVDO group at both studies (18,19).

The ADO is a biological process used for regenerating and consolidating bone between two bone segments generated after the osteotomies. These bone segments are gradually separated by distraction process. The osteogenic principles of the ADO are based in 3 phases: latency, activation and consolidation (5). There are different activation protocols of the distractor (27). First of all, the latency period around 5-7 days to permit healing of mucoperiosteum and reduce the risk of wound dehiscence. After that, distraction is achieved by activating the screw at a rate of 0.5-1 mm per day, followed by a consolidation period of 3-4 months after distraction (28,29).

The main advantages of the AVDO are predictability, the simultaneous grow of the soft tissues and the reduction of the treatment time compared to other techniques (4,8). The range of bone gain has been described between 5 and 15 mm (2).

The registered disadvantages of the AVDO are the need of the collaboration of the patients, programming more visits in the dental office and the costs. Additionally, complication rates range from 10 to 76%. The malposition of the distracted segment, the resorption of the distracted segment, the fracture of the distractor or basal bone, local infection and loss of vestibule are some of the most frequent complications (30-33).

The main distractors used for the AVDO are the extra-osseous or juxta-osseous distractors (EOD) and the intra-osseous distractors (IOD). The IOD pierces the bone segment to distracted, meanwhile in the EOD the bone segment is fixed with mini-plaques and monocortical screws at the buccal aspect (34).

Recent systematic reviews and meta-analysis have evaluated the different bone regeneration techniques for the treatment of the vertical bone atrophy. Elnayef *et al.* (35) concluded that the bone regeneration techniques that allow greater vertical bone gain were the inlay bone graft and the AVDO, but these techniques had higher complications rates in comparison with onlay bone graft and GBR. On the other hand, Camps-Font *et al.* (36) did not find statistically significant differences between distinct techniques for vertical mandibular atrophy in terms of implant and prosthesis failure rate, biologic complications, technical complications, patient preferences and peri-implant marginal bone loss. These investigators concluded which the short dental implants placement (5-8 mm) could be an alternative to bone regeneration techniques in order to restore a mandibular alveolar atrophy.

The choice of the bone regeneration technique must be done following the current best scientific evidence. According to the results of this systematic review, either techniques have shown similar outcomes in terms of implant survival and success rate. Thus, the surgeon must decide the treatment plan in accordance with the patient needs and opinions, considering the risks and benefits of each decision.

The inclusion of only 2 studies at high risk of bias with a small sample size limit the results of this systematic review. Furthermore, distinct dental implant and distractor brands used among the selected studies could influence the obtained results.

Both AVDO and ABG are effective bone regeneration techniques for the treatment of mandibular vertical bone atrophy.

In conclusion, a level B recommendation can be established for the use of AVDO for the treatment of the mandibular vertical bone atrophy. The authors recommend being cautious with the results of this study according to the limitations mentioned above. Long-term, well-designed randomized controlled trials comparing AVDO with distinct bone regeneration techniques such as the ABG and the GBR are needed.
